# Role of organic cation orientation in formamidine based perovskite materials

**DOI:** 10.1038/s41598-021-99621-1

**Published:** 2021-10-14

**Authors:** Siyu Liu, Jing Wang, Zhe Hu, Zhongtao Duan, Hao Zhang, Wanlu Zhang, Ruiqian Guo, Fengxian Xie

**Affiliations:** 1 Institute of Future Lighting, Academy for Engineering and Technology, Fudan University, Shanghai, 200433 China; 2 Institute for Electric Light Sources, School of Information Science and Technology, Fudan University, Shanghai, 200433 China; 3 Department of Optical Science and Engineering, Fudan University, Shanghai, 200433 China

**Keywords:** Theory and computation, Electronics, photonics and device physics, Solar energy

## Abstract

The rotation of organic cations is considered to be an important reason for the dynamic changes in stability and photoelectric properties of organic perovskites. However, the specific effect of organic cations rotation on formamidine based perovskite is still unknown. In our work, first-principles calculations based on density functional theory are used to examine the effect of the rotation of formamidine cations in FAPbI_3_ and FA_0.875_Cs_0.125_PbI_3_. We have comprehensively calculated the structure, electronic and optical properties of them. We found a coupling effect between formamidine cations rotation and cesium atom. This coupling effect changes the inclination angle of octahedron to regulate electron distribution, band gaps, and optical absorption. Hence, changing the cation orientation and substitution atom is a feasible way to dynamically adjust the energy band, dielectric constant and absorption edge of perovskite. Preparing perovskite with tunable properties is just around the corner through this way.

## Introduction

Since the concept of organic halide perovskite was proposed in 2013^1^, it has drawn substantial interests for researchers from materials, chemistry and physics field^2^. In 2015, Nam-Gyu Park et al.^3^ used 10% cesium to substitute formamidine cation and got lower trap-density, higher open circuit voltage, respectful thermal property and enhanced stability. Recently, as the efficiency of formamidine based perovskite materials has reached more than 20% efficiency and 3000 min T_80_ lifetime (the time that the solar cell can run at 80% of its initial performance)^4,5^, they have been considered as promising candidates for new era perovskite materials.

Though researchers try hard to promote efficiency and improve stability of formamidine based perovskite materials, the mechanism for improving the efficiency and stability of perovskite materials and devices is still not clear. For instance, how the photoelectric properties and charge dynamics in perovskites be affected by specific mechanisms including the size, the interaction of organic cations and the interaction with the crystal structure. Some former study thought that the main function of organic cations is to stabilize the structure and will not change photoelectronic properties^6^. Mehdizadeh et al.^7^ studied that the rotation of the MA in R_z_ and R_x_ modes causes substantial changes in the band structure, density of states, electron density, dielectric function, and absorption spectrum. They found that these changes are greatly affected by the interaction of cation–cation (MA-Pb) and cation–anion (MA-X) and van der Waals radius of organic cations. Researchers had also done related studies on the FA system and compared the differences from the MA system due to ion size, charge distribution and hydrogen bonding. Besides, the inorganic PbI_6_ octahedral frame experienced Glazer tilt with a time of 0.2–1.5 ps in MAPbI_3_, and the FA cation at A-site in FAPbI_3_ rotates or rolls in the cage (2 − 3 ps), which indicates organic cations’ orientation plays an important role in halide perovskite^8^. To be more specific, the orientation of organic cation has relations with ferroelectricity^9^, ion transport^10^, phase transition temperature^11^, water molecule adsorption and device stability^12^.

Recently, some researchers paid attention to this and used density functional theory (DFT) to describe this phenomenon in detail. Carlo et al.^13^ revealed the role of organic cations in hybrid halide perovskite MAPbI_3_. Furthermore, Jonathon et al.^14^ considered the influence of the rotation of the MA cation along the coordinate axis and the coaxial precession on the performance of perovskite. Besides, Nathaniel et al.^15^ considered the coupling effect between cations and inorganic lattice, the different energy barrier between FA and MA cation rotation. Akhtarianfar et al.^16^ investigated the formamidine cation rotation in FAPbI_3_. But there is no one has studied the effect of cation orientation on inorganic ions, perovskite lattice and properties in the FA/Cs system of formamidine based perovskite. On the other hand, studies show that the rotation of cations decreases at low temperature and high pressure^17,18^, so it is possible to control the synthesis of cation-oriented cubic perovskite to bring vital different changes in this field.

**Figure 1:**
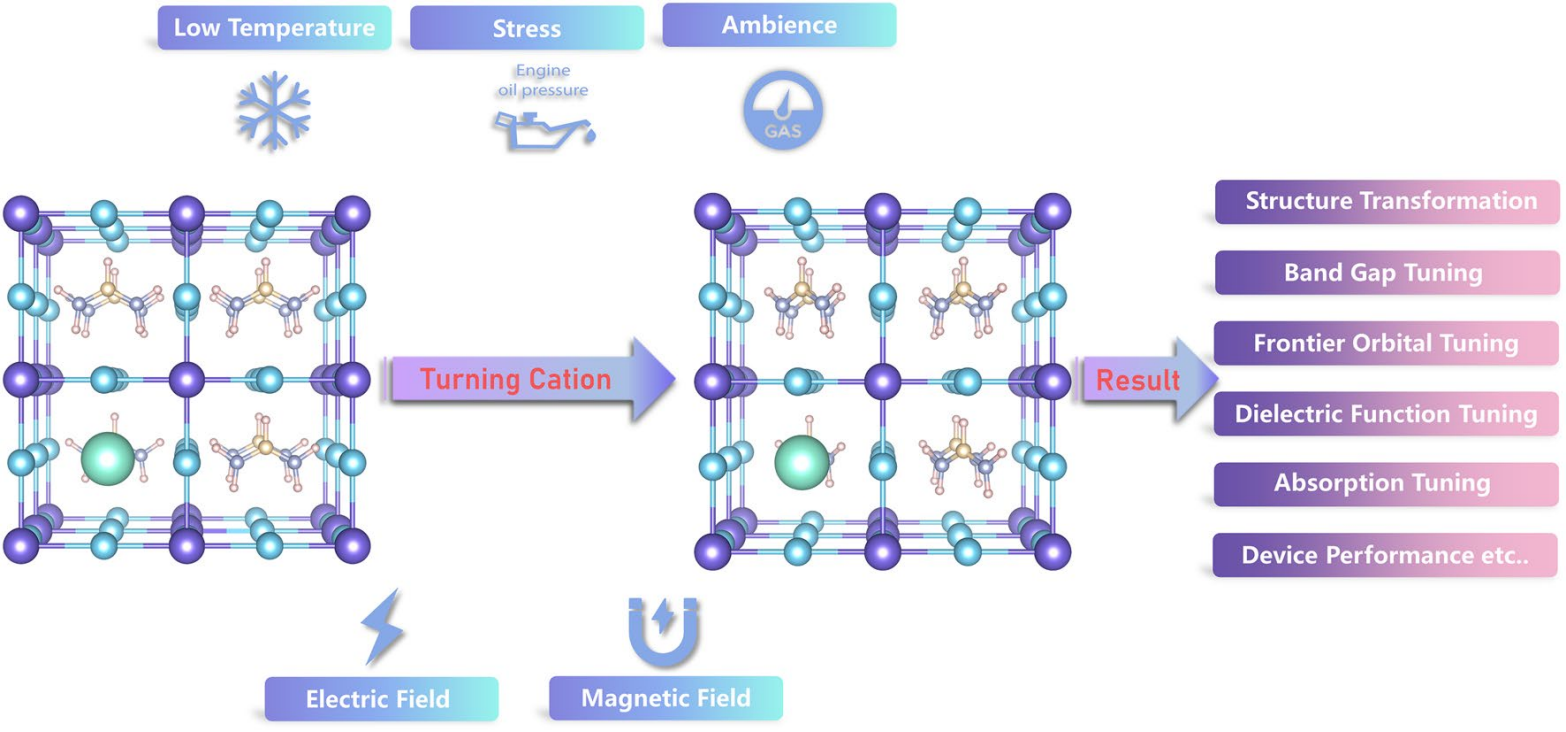
Schematic diagram of possible ways to change the orientation of organic cation and the result lead by it.

Hence, our work is the first time to investigate the complex coupling effect of organic orientation in formamidine based perovskite. We used DFT theory to build a supercell to study this problem, and tried to build a reasonable explanation of it. We found that the coupling effect of organic cations and cesium atom affected many properties of this mixing system, such as lattice constant, band structures, charge distribution, dielectric constant, optical absorption properties, and even the performance of solar cell device (Fig. 1). Our work not only expounds the mechanism of the role of cation orientation in the mixed system, but also points out a feasible way for the synthesis of electron and optically adjustable perovskite materials and devices.

## Computational details

All of these first-principles calculations were performed by using the Vienna Ab initio Simulation Package (VASP)^19,20^. Perdew–Burke–Ernzerhof (PBE) exchange correlation functional was used within a generalized gradient approximation (GGA)^21^ and optB86b^22^ van der Waals force revision. We focused on the pseudo-cubic phase of formamidine based perovskite with the chemical structures of FAPbI_3_ and FA_0.875_Cs_0.125_PbI_3_. A cut-off energy of 400 eV was chosen to reach the desired accuracy. For the optimization of the geometric structure, the total energy convergence criterion was chosen as 10^–5^ eV, and the force on each atom was converged to an accuracy of 0.01 eV·Å^−1^. The size of the FAPbI_3_ supercell is 2 × 2 × 2, and one formamidine is substituted by a cesium atom to simulate the 12.5% doping. A reciprocal-space sampling with gamma center in an 8 × 8 × 8 Monkhorst–Pack k-point mesh^23^ was set in the Brillouin zone for the standard DFT calculations. The occupied and empty bands set in the DFT calculation are 240 for FAPbI_3_ and 224 for FA_0.875_Cs_0.125_PbI_3_. Molecular dynamics simulation used canonical ensemble (NVT). The temperature of simulation is 400 K. The total simulation time of each trajectory was 5 ps with a 2 fs timestep. Considering the computational accuracy and the enormous demands of calculation resources, the phase of perovskite will change from high to low temperature, and VASP calculations are performed at 0 K, we did not calculate phonon spectrum and elastic constants. And to mitigate the effect due to the 0 K calculation, we used the crystal constant from experiment as the basis to build our model. The VESTA software was used to visualize crystal structures and electron distribution^24^. The VASPKIT toolkit was used to post-process the band gap, density of state and dielectric constant data^25^.

For calculating cohesive energy ${E}_{c}$, the formula is as follows:

1 $${E}_{c}=\frac{E\left({A}_{m}{B}_{n}{C}_{p}{D}_{q}\right)-mE\left(A\right)-nE\left(B\right)-pE\left(C\right)-qE\left(D\right)}{The\, number\, of \,atoms \,in \,a \,conventional \,unit \,cell}$$

where $E({A}_{m}{B}_{n}{C}_{p}{D}_{q})$ means the relaxed energy of structure, $A, B, C, D$ are the elements in structure, $m, n, p, q$ are the numbers of each element. $E\left(A\right), E\left(B\right), E\left(C\right), E(D)$ are the energy of single atom of each element.

For evaluating the optical properties, we calculated the frequency-dependent complex dielectric function^26^.

2 $$\varepsilon \left(\omega \right)= 1+ \frac{8\pi }{\Omega {N}_{k}}\sum_{k,v,c}\frac{{\left|\langle {\varphi }_{kv}\left|\widehat{v}\right|{\varphi }_{kc}\rangle \right|}^{2}}{{\left({E}_{kc}-{E}_{kv}\right)}^{2}\left({E}_{kc}-{E}_{kv}-\omega -i\eta \right)}$$

where $\Omega$ is the volume of the cell, $\widehat{v}$ is the operator of velocity,$\eta$ is an opportune broadening factor, ${N}_{k}$ is the total number of k-points in the Brillouin zone, and the indices $c$ and $v$ show the unoccupied and occupied states, respectively. The frequency-dependent absorption coefficient $\alpha \left(\omega \right)$, is given by

3 $$\alpha \left(\omega \right)= \omega \sqrt{\frac{-\mathrm{Re}\varepsilon \left(\omega \right)+ \sqrt{{\mathrm{Re}}^{2}\varepsilon \left(\omega \right)}+{\mathrm{Im}}^{2}\varepsilon \left(\omega \right)}{2}}.$$

## Results and discussion

### Structural stability and adjustment

Figure S1 shows the initial 2 × 2 × 2 supercell of FAPbI_3_ and FA_0.875_Cs_0.125_PbI_3_ with formamidine cations facing the (100), (110) and (111) direction. Before all others, all the calculations have converged on the energy convergence standard 10^–5^ eV and the force convergence standard 0.01 eV·Å^−1^ per atom by using GGA-PBE exchange correlation functional^21^ with van der Waals force correlation^22^ without considering the spin–orbit coupling (SOC). We also calculated cohesive energy and ab-initio molecular dynamics (AIMD) in 400 K to confirm the dynamical stability of these hybrid perovskites^27,28^. The cohesive energy of all our structures is less than zero, which means these structures are stable in 0 K. The energy-time curves of AIMD of all our structures are straight, which means these structures are stable in 400 K (Table 1; Figure S2).

**Table 1:** Calculated lattice constant, cell volume, distortion index (bond length) and relaxed energy of FAPbI_3_ and FA_0.875_Cs_0.125_PbI_3_.

System	Orientation	Lattice constant (Å)	Cell volume (Å^3^)	Distortion index (bond length)	Relaxed energy (eV)	Cohesive energy (eV)
FAPbI_3_	(100)	12.6211	2010.4645	0.00935	− 340.5251	− 3.4751
	(110)	12.4713	1939.6834	0.00701	− 340.8722	− 3.4787
	(111)	12.5371	1970.5563	0.01219	− 340.6713	− 3.4767
FA_0.875_Cs_0.125_PbI_3_	(100)	12.6140	2007.0482	0.01096	− 301.7401	− 3.3029
	(110)	12.4839	1945.5874	0.00764	− 301.9107	− 3.3048
	(111)	12.5377	1970.8478	0.00938	− 301.7699	− 3.3032

**Figure 2:**
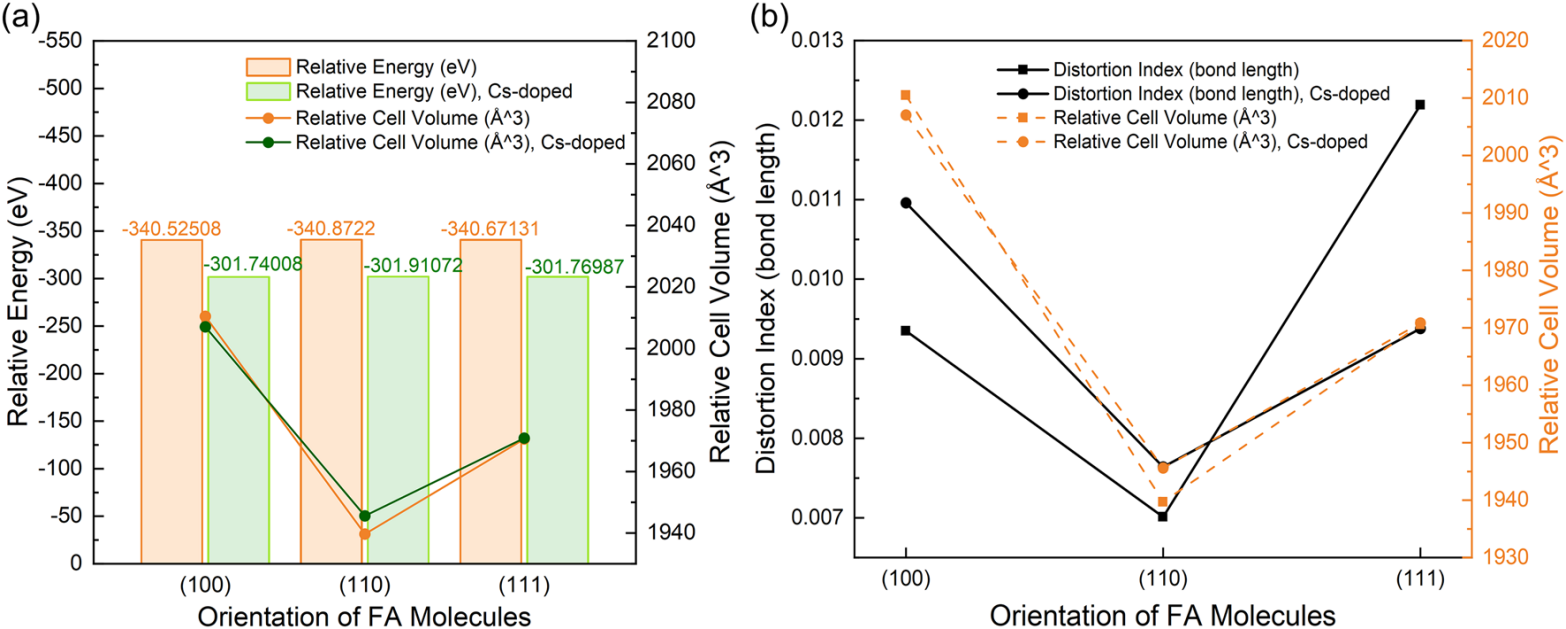
(**a**) Relative energy and relative cell volume in different FA cations orientation; (**b**) relative energy and distortion index (bond length) in different FA cations orientation.

In Table 1 and Fig. 2, the data of relaxed structures for the cubic phase of FAPbI_3_ and FA_0.875_Cs_0.125_PbI_3_ are displayed. Refer to previous research, PBE-vDW method can obtain the most accurate lattice constant than DFT-PBE and HSE06 methods in formamidine halide perovskite^29^. The lattice constant of FAPbI_3_ supercell we calculated in (100) direction is close to antecedent experiment^30,31^ and calculation^29^. We used the cell volume and lattice constant obtained by the cube root of cell volume for measuring the disorder of the structure due to the structure will turn from cubic phase to pseudo-cubic phase^32^. Therefore, we have reason to believe that our model effectively represents the specific case of an actual condition of perfectly aligned molecular dipoles.

Firstly, let us separately discuss changes lead by orientation in FAPbI_3_ and FA_0.875_Cs_0.125_PbI_3_ system. From Fig. 2a, we can discover that the relaxed energy at each formamidine orientation is similar, and the energy barrier is 0.2–0.3 eV for each condition. It’s not a tremendous energy barrier, so formamidine cations can freely rotate in cage. According to previous research, the formamidine cations are indeed rapid rotating with a time period of 2–3 ps and PbI_6_ octahedral framework is undergoing Glazer tilting with a time period of about 0.2–1.5 ps^17,33^.

Besides the relaxed energy, the relative cell volume of (110) is the smallest in three rotation models, leading to the minimum of lattice constant. The change of lattice constant in FAPbI_3_ system is affected by the reorientation of formamidine cations because their atom arrangement and charge distribution are anisotropic. These factors cause Pb–I–Pb to be subject to different forces like N–H···I hydrogen bond and van der Waals force in different directions^34^. Furthermore, the contraction of perovskite lattice from FAPbI_3_ to FA_0.875_Cs_0.125_PbI_3_ is usually attributed to the substitution from formamidine to cesium because of the smaller radius of cesium^35^. In fact we indeed find that the lattice constant of FA_0.875_Cs_0.125_PbI_3_ in (100) direction has a 0.1% decrease than FAPbI_3_, which is consistent with the experiment^36^. But the phenomenon in (110) and (111) is different. This difference can be attributed to the coupling effect between the orientation of organic cations and the addition of cesium.

This coupling effect also influences the tilt of PbI_6_ octahedron. From Fig. 2b, both FAPbI_3_ and FA_0.875_Cs_0.125_PbI_3_ systems get the minimum value of distortion index (this index describes the degree of tilt of PbI_6_ octahedron) in (110) direction. We can also directly see the degree of deformation in Figure S3. The difference is that FAPbI_3_ gets the maximum value in (111) direction but FA_0.875_Cs_0.125_PbI_3_ gets it in (100) direction, which maybe lead by the cesium and formamidine cations co-effect.

All in all, the reorientation of formamidine cations and the entrance of cesium give the formamidine perovskite adjustment structural properties. Lots of researches before shows that organic cations will be ‘frozen’ in low temperature^14,37,38^. And the structural stability is also be closely bound up with the internal structure of perovskite. Therefore, our work indicate which low temperature state is good for the application in different areas.

### Electronic properties

**Figure 3:**
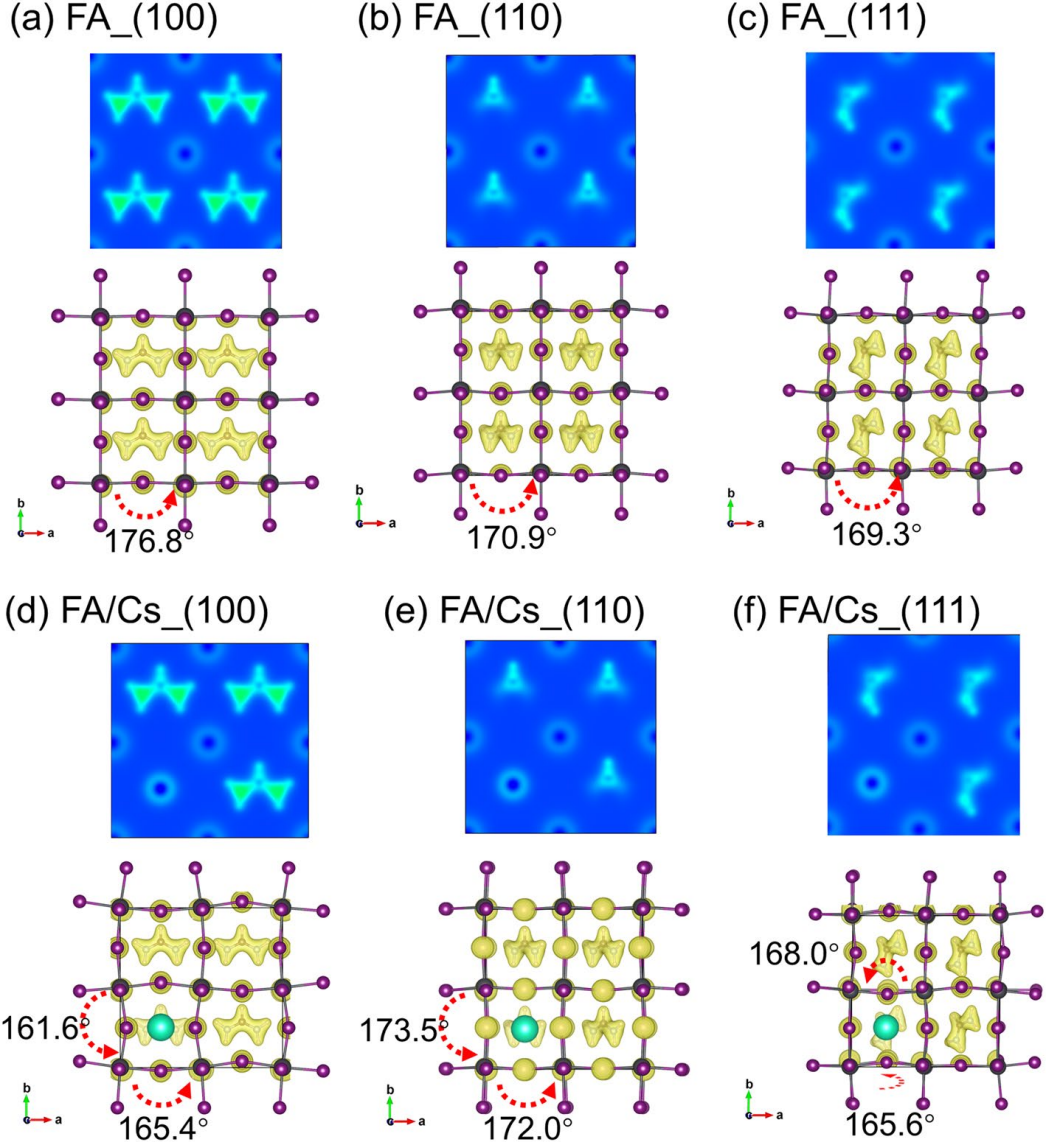
Two-dimensional (2D) depiction of electron charge density in crystallographic plane of (001) in FAPbI_3_ (**a**) (100), (**b**) (110), (**c**) (111) and FA_0.875_Cs_0.125_PbI_3_, (**d**) (100), (**e**) (110), (**f**) (111). 3D charge map is also shown for each sub-figure.

Figure 3 shows the 2D and 3D maps of electron charge density. This map reveals the interaction among the components of the perovskite and how electrons distribute in the lattice. We set the isosurface level 0.1 electrons/bohr^3^ to see the distribution more directly.

According to the non-doped FAPbI_3_ perovskite, the interaction of formamidine with PbI_6_ octahedron leads to a considerable disorder, which alters its volume (cell volume is changed as well) (refer to Table 1; Fig. 2). Interestingly, Table 1 tells us the distortion of PbI_6_ octahedron reaches a minimum in (110) direction. This phenomenon can be attributed to that the symmetrical interactions in (110) plane uniformly inflate the size of PbI_6_, which leads to a decrease of PbI_6_ distortion degree compared with other orientations^7^.

Moreover, the FA_0.875_Cs_0.125_PbI_3_ system shows some similarities and differences. Diverse directions indeed tilt the PbI_6_ octahedron and Pb–I–Pb bond angle, but the addition of cesium also affects them. It’s obviously that the cesium atom shows strong ionization because the light around the cesium atom is weak in 2D contour plot Fig. 3d–f. This is because the electronegativity of cesium is near the largest in periodic table, and the electron in Cs 6s orbit is easy to be seized and lose. Owing to this, the bond angle of Pb–I–Pb is strongly tilted when formamidine cations orient to (100) direction. In (110) direction, the distortion reduces than (100) orientation, and we can see the bond angle of Pb–I–Pb is tilting slightly than (100) orientation. This reduction maybe is the same reason like FAPbI_3_ we discussed below. But the FA_0.875_Cs_0.125_PbI_3_ system’s distortion index is larger than FAPbI_3_ system when orients to (110) direction, which is caused by the smaller radius of cesium. When turns to (111) direction, the Pb–I–Pb bond occurs inner fold on x–y plane with a 7.8° angle but the distortion is smaller than FAPbI_3_ in the same direction. This is because the substitution of cesium reduces the charge interaction lead by (111) direction of formamidine cations. To be more specific, the (111) orientation of formamidine causes a catercorner electronic interaction, but cesium atom also possesses a strong polarization force. The coupled effect of them leads to this result, and even affects the bandgap and density of state we will discuss after.

**Figure 4:**
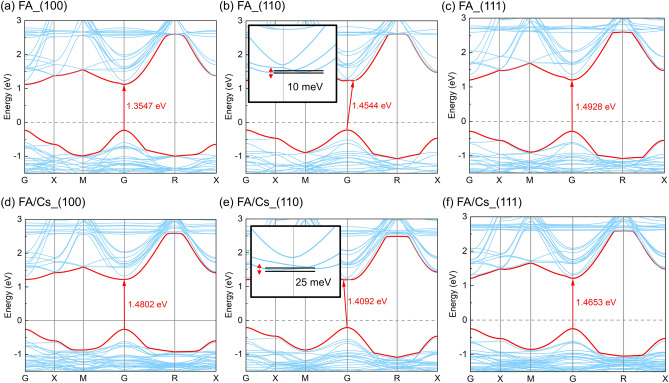
(**a**–**c**) Band gap of FAPbI_3_ with different FA cations orientation: (**a**) (100), (**b**) (110), (**c**) (111); (**d**–**f**) band gap of FA_0.875_Cs_0.125_PbI_3_ with different FA cations orientation: (**d**) (100), (**e**) (110), (**f**) (111).

**Figure 5:**
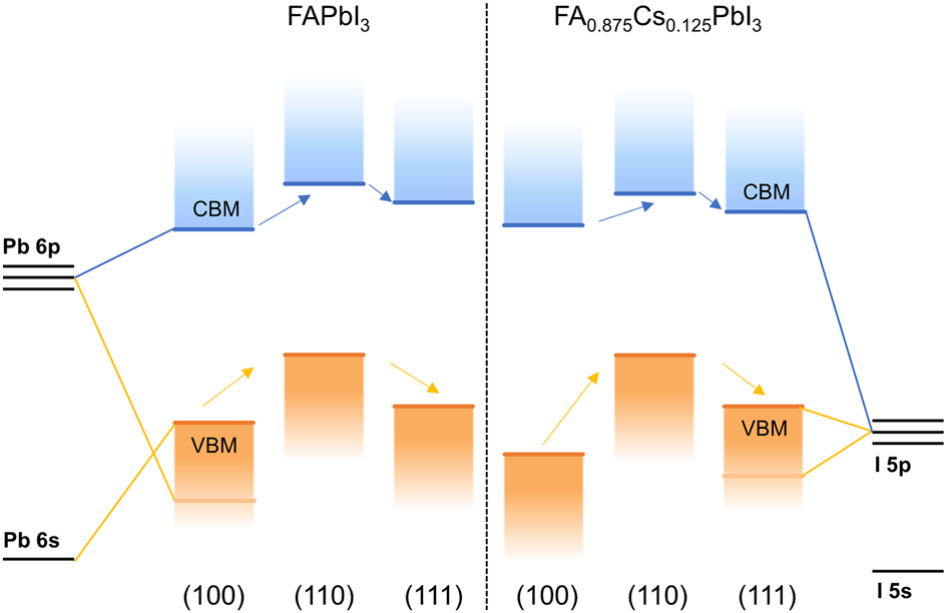
Schematic energy level diagram of different formamidine orientations (black line: atom energy level; blue line: valence band minimum; orange line: conduction band maximum; black dotted line: fermi energy).

**Figure 6:**
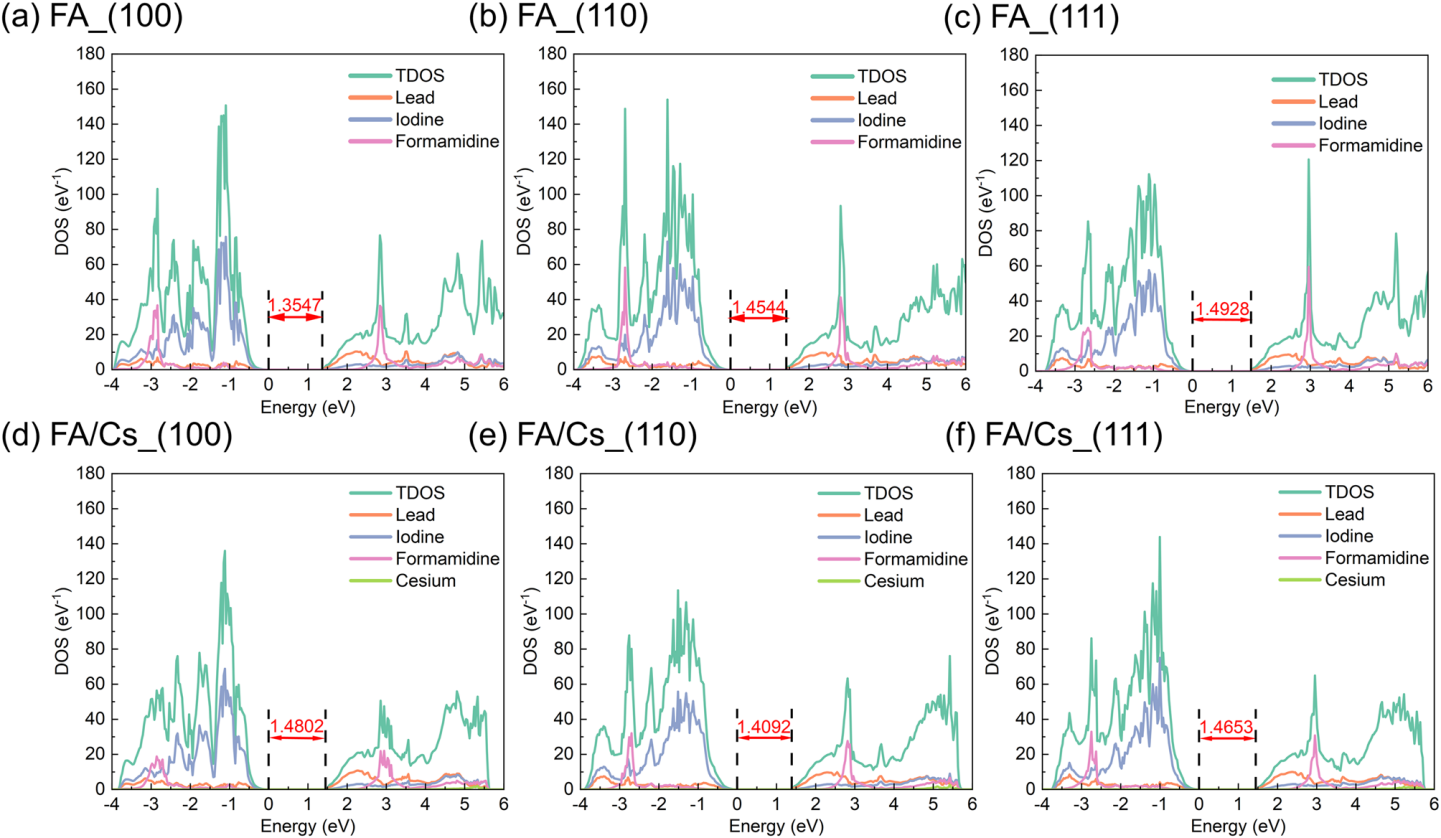
Total density of state (TDOS) and projected density of state (PDOS): (**a**–**c**) FAPbI_3_ with different FA cations orientation: (**a**) (100), (**b**) (110), (**c**) (111); (**d**–**f**) FA_0.875_Cs_0.125_PbI_3_ with different FA cations orientation: (**d**) (100), (**e**) (110), (**f**) (111).

Now let’s focus on the role of formamidine orientation in electronic structure. Figures 4, 5 and 6 shows the band structure and density of state (DOS) of formamidine based perovskite. Figure 4 indicates the trends of band gap of FAPbI_3_ and FA_0.875_Cs_0.125_PbI_3_. Since spin–orbit coupling (SOC) will change the band gap, and GGA will underestimate the band gap, the effects of them coincidentally make the band gap calculated without considering the SOC close to the experimental value^32,34,39^. While using HSE06 + SOC can get a more accurate value, we just want to focus on the variation of band gap. Hence, it’s enough for us to use the result of PBE-vDW function.

From Fig. 4, we can see that the band gap of FAPbI_3_ increases from (100) to (110) and to (111) direction, while the band gap of FA_0.875_Cs_0.125_PbI_3_ decreases first and then increases. In both models, the band gap in the (110) direction changes from direct to indirect. We propose that the reasons for these phenomena may be as follows: (1) the distortion of PbI_6_ octahedron; (2) the addition of cesium atom; (3) the dispersive force between the organic cations and the inorganic framework.

First and foremost, the lattice contraction is affected by the A-site cation radius. If the perovskite lattice contracts, the band gap will increase. That’s why the band gap of FA_0.875_Cs_0.125_PbI_3_ will increase from 1.3547 eV of FAPbI_3_ to 1.4802 eV in (100) direction. Moreover, metal-halide orbital overlap is a σ* interaction, decreasing the Pb–I–Pb bond angle will reduce the degree of overlap between the metal and halide orbitals^40^. The result is that when cesium substitution increases, the valence band moves to a slightly lower energy (see in Fig. 5; Table S1), thereby widening the band gap. This phenomenon is consistent with the Cs-doped experiment^3,35,36^.

Secondly, the tilt of the PbI_6_ octahedron can change the spatial distribution and overlap of electron orbits, and this tilting effect depends on the electronic interaction between ions. This condition is well known as the dipole coupling effect lead by the number of ^1^H-^1^H dipolar couplings on adjacent cations^41^. From Table S1 and Fig. 4b,c, the band gap increases though the lattice constant contract, which can be attributed to the turning of formamidine cations from (110) to (111) direction enhances the dipole–dipole interaction and the distortion index. Meanwhile, from Table 1 and Fig. 4d–f, the smaller size of cesium and the largest distortion index increase the band gap in (100) direction. But the reorientation decreases the distortion index, leading to the reduction of band gap in (110) and (111) directions. Besides, the addition of cesium decreases the number of N–H···I hydrogen bond^14,33^, and this influence may enlarge in (110) and (111) directions. Hence, the band gap reduces and even is smaller than the FAPbI_3_ system in these two directions.

Thirdly, the dispersive force between the organic cations and the inorganic framework leads to the gap transformation from direct to indirect in (110) direction of both systems (see in Fig. 4b,e). Carlo et al.^13^ firstly discovered and demonstrated this change when viewing the turning of methylamine cations in MAPbI_3_. Then Farshad et al.^16^ also found this phenomenon in FAPbI_3_. Besides, the static effects, such as Rashba splitting in the conduction band may play an effective role in VBM^42^. At our work, we also observed 10 meV and 25 meV departure of VBM in (110) direction of FAPbI_3_ and FA_0.875_Cs_0.125_PbI_3_. This change may have a potential impact on the transport and recombination of carriers.

From Fig. 5, we can see that the orientation of formamidine cations not only affect the band gap, but also turn the VBM, CBM and Fermi energy. They are rising first and then falling in general and the trend is accordant in both systems. Recently, band gap engineering is popular^40,43,44^. Researchers achieved high efficiency by turning perovskite band gap alignment with ETL or HTL layer. If we can control the band gap by turning the intrinsic organic cation, it’s a new idea to achieve higher carrier mobility, J_sc_, V_oc_ and efficiency.

As is well-known that the CBM is mainly contributed by the electrons of the Pb 6p orbitals, and the VBM mainly is contributed by the electrons of I 5p orbitals. From the PDOS result in Fig. 6, we indeed find that the frontier orbitals are composed of Pb atom and I atom.

In addition, the difference between energy state below and above the Fermi energy means the bang gap, and it’s consistent with the result of band gap in Fig. 4. Besides, comparing Fig. 6a–c and Fig. 6d–f, the addition of cesium atom makes the total DOS distribution more localized around the Fermi energy, which enhances ionic interaction though the electrons of cesium do not distribute in frontier orbitals (Fig. 6d–f). This is thought that the addition of cesium makes perovskite structure more stable^8^. Moreover, the turning of orientation from (100) to (111) in Fig. 6a–c makes the formamidine density of state in about 3 eV deep conduction band stronger. This condition is the same with Cs-doped system. This confirms the interaction between formamidine and PbI_6_ framework. Compared with the same direction of FAPbI_3_ and FA_0.875_Cs_0.125_PbI_3_, cesium atom exists in deeper conduction bands, and the energy state distribution is deep than 5 eV. Cesium ion exhibits strong ionization and this may contribute to other atoms’ charge distribution in space and the stability of the structure.

### Optical properties

**Figure 7:**
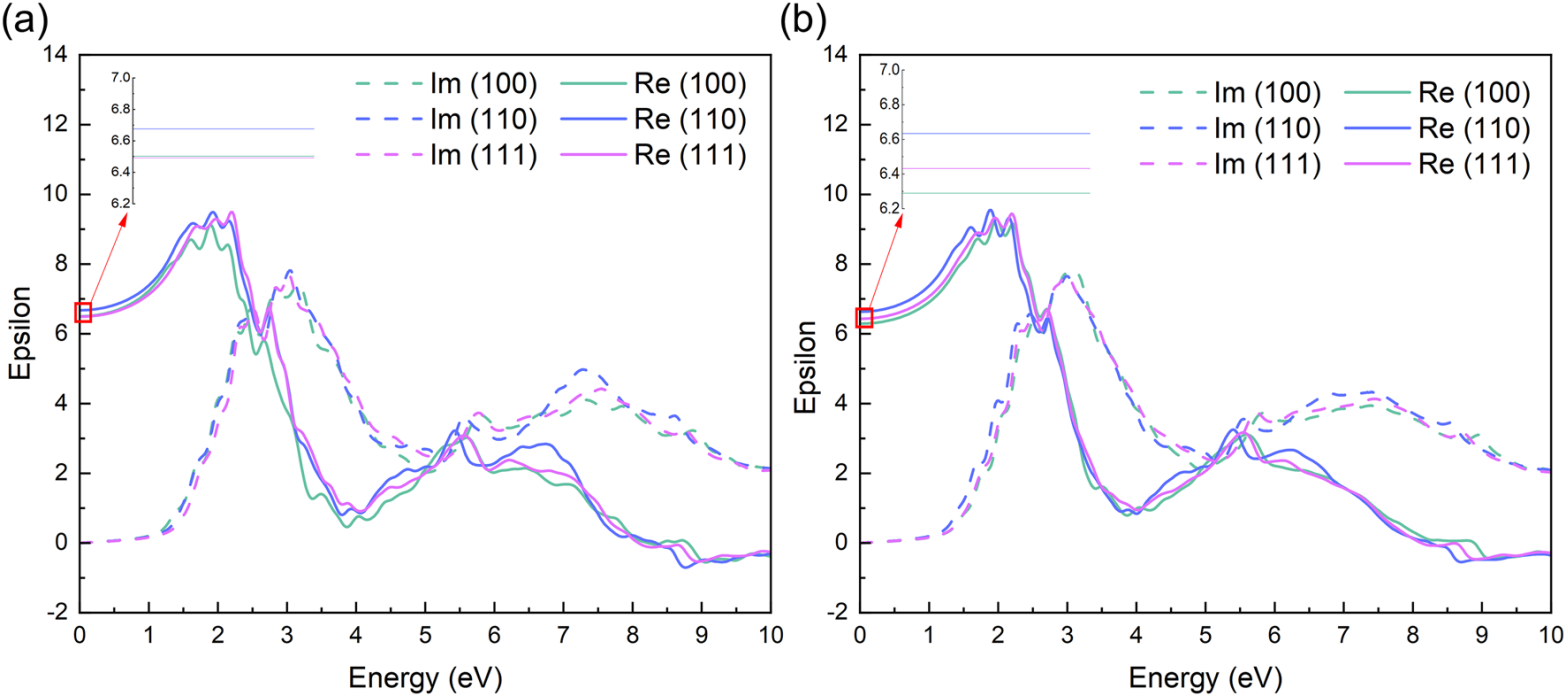
The real and imaginary part of dielectric function of (**a**) FAPbI_3_ and (**b**) FA_0.875_Cs_0.125_PbI_3_ with different FA cations orientation.

**Figure 8:**
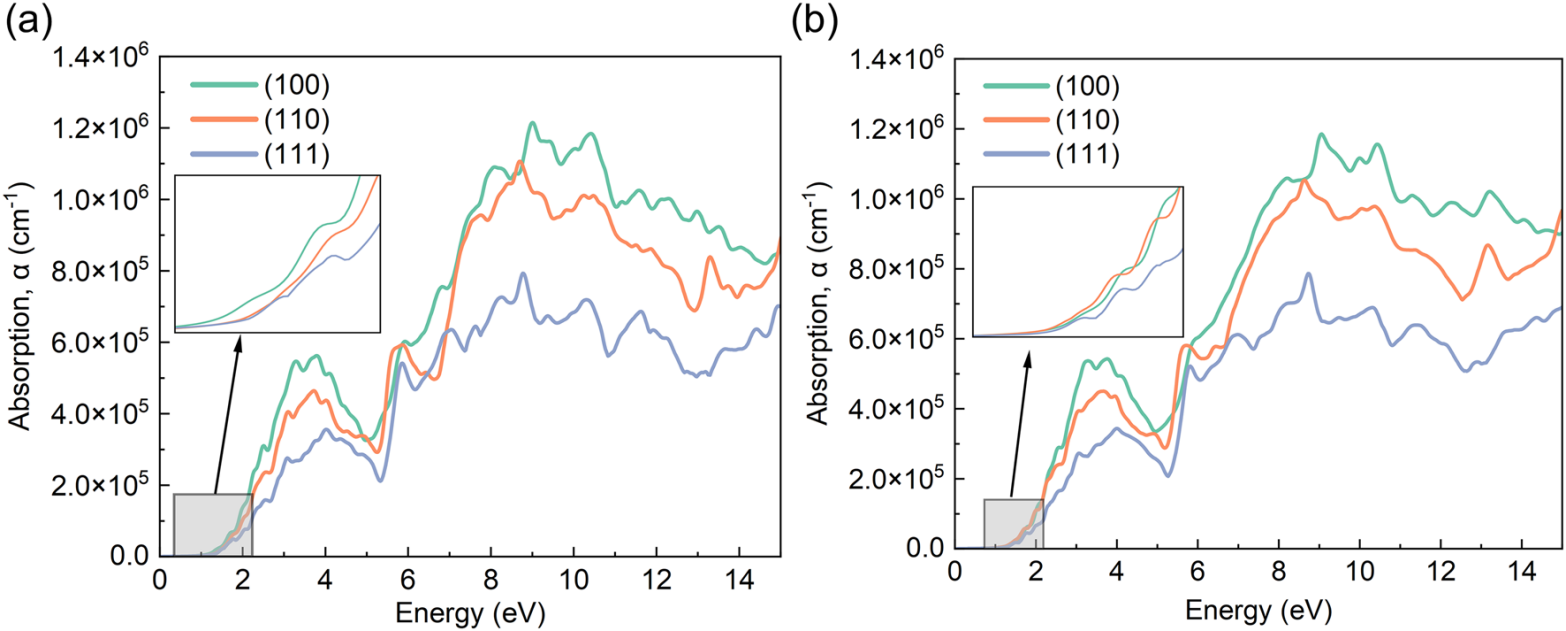
Optical adsorption spectrum of perovskite structures of (**a**) FAPbI_3_ and (**b**) FA_0.875_Cs_0.125_PbI_3_ with different FA cations orientation.

With the special electronic structure, FAPbI_3_ also has fantastic ability of optical absorption in the visible range used in solar cell and light emitting diode. Many features of FAPbI_3_, such as excellent stability under high humidity, sharper absorption edge, high charge mobility and low exciton binding energy can be attributed to the high dielectric constant of the material^45^. The coupling effect of organic cation orientation and the substitution of cesium can also be observed in optical properties. The reorientation of the organic cation and the addition of cesium with their correlated dipole moment dedicates to the dielectric response and throw a crystal field to formamidine based perovskite^7^. To study the role of organic cation with cesium atom, we calculated dielectric function in Fig. 7 and absorption spectrum in Fig. 8 for FAPbI_3_ and FA_0.875_Cs_0.125_PbI_3_ in (100), (110) and (111) direction.

Figure 7 shows the real and imaginary part of dielectric function. Generally speaking, the real part of dielectric function represents static dielectric constant at E = 0, which is calculated to be 6.50, 6.68 and 6.49 for FAPbI_3_ and 6.29, 6.63 and 6.43 for FA_0.875_Cs_0.125_PbI_3_ in (100), (110) and (111) direction (inset zoomed pictures of Fig. 7; Table S2). It’s obviously that the rotation of formamidine cations change the static dielectric constant in different orientation, and the entrance of cesium contracts the value of static dielectric constant compared with FAPbI_3_. This transformation definitely influences the optical properties like absorption in Fig. 8. The dashed curves in Fig. 7a,b display imaginary part of dielectric function. And it’s well known that the first peak represents as the direct optical transition, which is also called optical gap^7^. It can be seen in Fig. 7a that the rotation of formamidine cations from (100) to (111) leads to a blue shift in FAPbI_3_. But a red shift occurs when formamidine cations rotate from (100) to (111) in FA_0.875_Cs_0.125_PbI_3_. This is as well as the trends of band gap and absorption spectrum.

From Fig. 8, the inset zoomed pictures show band edge absorption condition, which has a strong correlation with band gap. All of these same trends prove the orientation of formamidine cations and the addition of cesium exist coupling effect. Besides, the after peak sometimes is thought as the exciton emission peak in quantum dot perovskite^46^.

Hence, the coupling effect of organic cation orientation and the substitution of cesium makes the optical performance of the mixed cation halide perovskite tunable. This is a crucial and promising method for manufacturing light-absorbing devices with variable intrinsic properties.

## Conclusions

Today, the efficiency and stability of formamidine based perovskite materials have been rapidly improved, but there are still many unsolved mysteries in the theories used to explain its advantages over other materials. Our work suggests that the size of PbI_6_ octahedron, lattice constant, charge distribution, band gap, density of state and absorption spectrum are inseparable from the orientation of organic cations and its coupling with cesium atom.

To be more specific, when the cation rotates from (100) to (110) and then to (111), the lattice constant and distortion index of FA and FA/Cs systems both decrease first and then increase. Although the minimum point is the same, the distortion index of the (111) plane in the FA/Cs system is abnormally smaller than the FA system due to the interaction between FA orientation and Cs. These results are due to the orientation of cations and the addition of cesium atom, which change the forces between ions in the crystal lattice, such as van der Waals forces and N–H···I hydrogen bonds.

In regard to electronic and optical properties, the different orientations of FA lead to changes in the band gap. Particularly, in the (110) direction, the band gap of the formamidine based perovskite becomes indirect. The addition of Cs also changes the band gap. We summarize the reasons into the following three points: (1) the distortion of PbI_6_ octahedron; (2) the addition of cesium atom; (3) the dispersive force between the organic cations and the inorganic framework. Moreover, the orientation of FA changes the distribution of electrons in the frontal orbit, while the addition of Cs makes it more localized and concentrated near the band gap. Furthermore, the orientation of FA changes the energy level of CBM, VBM and Fermi energy, which can provide a new idea for the experiment of band gap adjustment and alignment. Eventually, the orientation of FA and the doping of Cs also change the dielectric constant and the peak of the optical absorption spectrum.

Overall, this work starts from the formamidine rotation and its interaction with cesium atom. We demonstrate a possible way to engineer lattice constant, band gap, density of state and absorption spectrum, which is a prerequisite for controllable variable high-efficiency optoelectronic device.

## Supplementary Information

Supplementary Information.

## Data Availability

The datasets generated during and/or analyzed during the current study are available from the corresponding author on reasonable request.
