# Supplementary material for: Role of organic cation orientation in formamidine based perovskite materials
Source: Sci Rep. 2021 Oct 14;11:20433. doi: 10.1038/s41598-021-99621-1 (PMC8517011; doi:10.1038/s41598-021-99621-1)
Supplement: Supplementary file 1 — Supplementary Information. [file 41598_2021_99621_MOESM1_ESM.pdf]

# Supplementary Information

Siyu Liu[1], Jing Wang[2], Zhe Hu[2], Zhongtao Duan[1], Hao Zhang[3], Wanlu Zhang[2], Ruiqian Guo[1,2*], Fengxian Xie[1,2*]

1. Institute of Future Lighting, Academy for Engineering and Technology, Fudan University, Shanghai 200433, China.
2. Institute for Electric Light Sources, School of Information Science and Technology, Fudan University, Shanghai 200433, China.
3. Department of optical science and engineering, Fudan University, Shanghai 200433, China
Correspondence and requests for materials should be addressed to R.Q.G (email: rqguo@fudan.edu.cn) and F.X.X (email: xiefengxian@fudan.edu.cn).

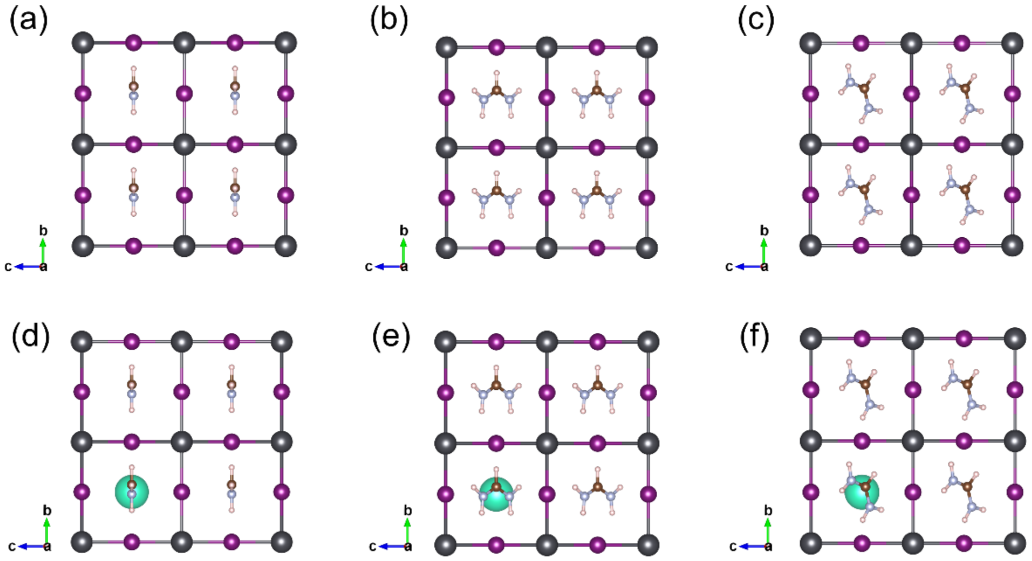


Figure S1. Original 2×2×2 supercell structure of the cubic phase $FAPbI_3$ and $FA_{0.875}Cs_{0.125}PbI_3$, and formamidine cations face (a, d) (100) (b, e) (110) (c, f) (111) direction (Purple: Iodine, Grey: Lead, Dark brown: Carbon, Celeste: Nitrogen, Light brown: Hydrogen, Green: Cesium)

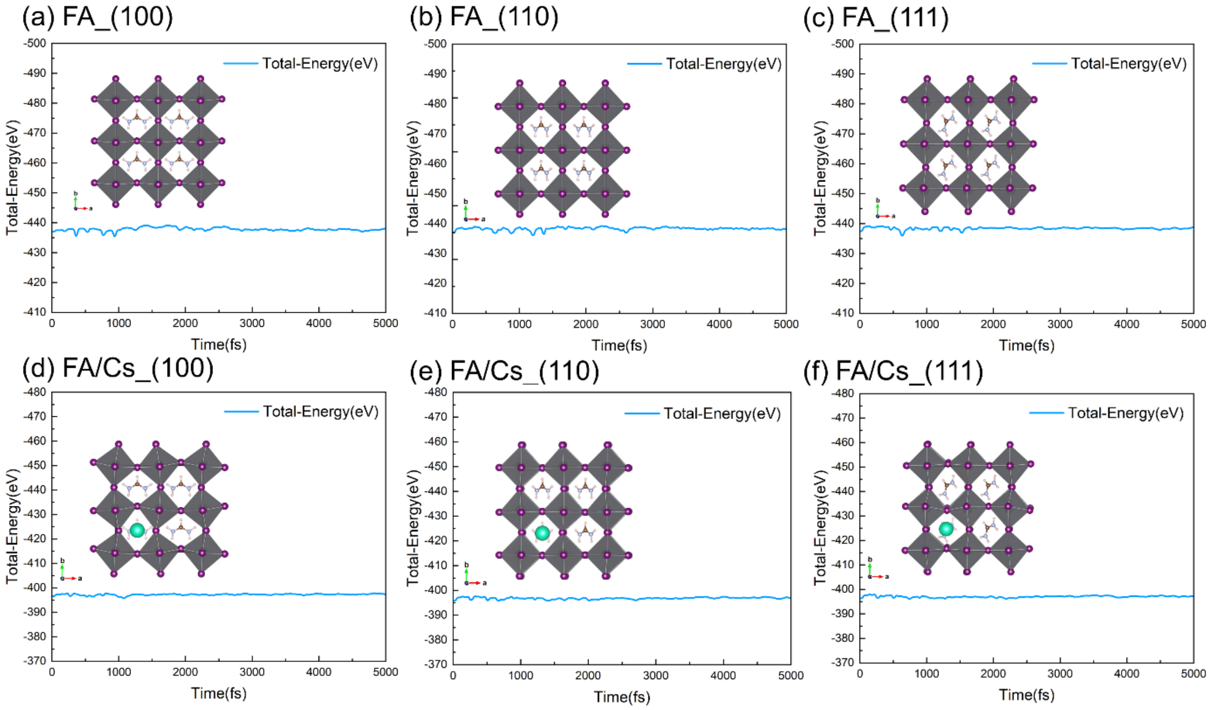


Figure S2. The results of AIMD: (a-c) Energy variation with temperature of $FAPbI_3$ with different FA cations orientation: (a) (100) (b) (110) (c) (111); (d-f) Energy variation with temperature of $FA_{0.875}Cs_{0.125}PbI_3$ with different FA cations orientation: (d) (100) (e) (110) (f) (111)

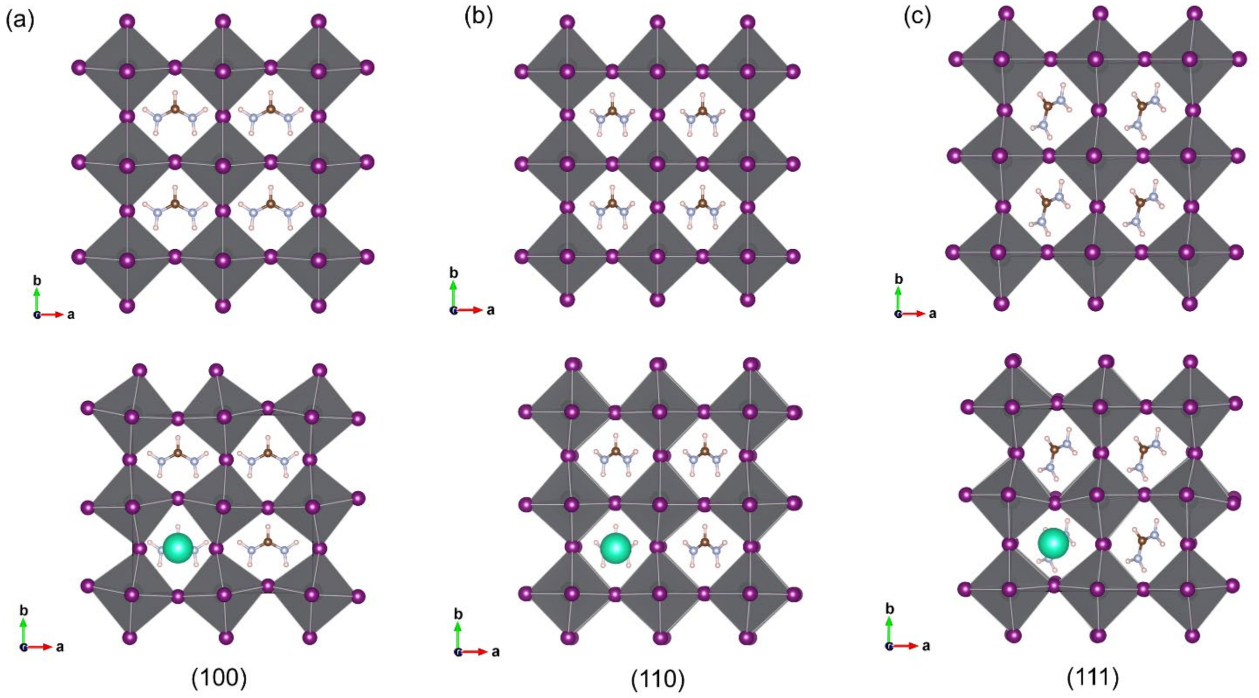


Figure S3. Relaxed structure of $FAPbI_3$ (upper pictures) and $FA_{0.875}Cs_{0.125}PbI_3$ (lower pictures) in different orientation:(a) (100) (b) (110) (c) (111)

Table S1.The fermi energy, valence band maximum, conduction band minimum, band gap and band type in (100), (110) and (111) directions for $FAPbI_3$ and $FA_{0.875}Cs_{0.125}PbI_3$

| System | Orientation | Fermi Energy (eV) | VBM (eV) | CBM (eV) | Band gap (eV) | Band type |
|---|---|---|---|---|---|---|
| $FAPbI_3$ | (100) | 1.7646 | 1.5292 | 2.8840 | 1.3547 | Direct |
| | (110) | 1.9348 | 1.7090 | 3.1634 | 1.4544 | Indirect |
| | (111) | 1.8380 | 1.5488 | 3.0416 | 1.4928 | Direct |
| $FA_{0.875}Cs_{0.125}PbI_3$ | (100) | 1.6885 | 1.4300 | 2.9102 | 1.4802 | Direct |
| | (110) | 1.9119 | 1.7017 | 3.1109 | 1.4092 | Indirect |
| | (111) | 1.7947 | 1.5415 | 3.0068 | 1.4653 | Direct |

Table S2.Static dielectric constant in (100), (110) and (111) directions for $FAPbI_3$ and $FA_{0.875}Cs_{0.125}PbI_3$

| System | Orientation | Static Dielectric Constant |
|---|---|---|
| $FAPbI_3$ | (100) | 6.50 |
| | (110) | 6.68 |
| | (111) | 6.49 |
| $FA_{0.875}Cs_{0.125}PbI_3$ | (100) | 6.29 |
| | (110) | 6.63 |
| | (111) | 6.43 |
